# Short-term temporal analysis and children's knowledge of the composition of important medicinal plants: the structural core hypothesis

**DOI:** 10.1186/s13002-022-00548-2

**Published:** 2022-07-09

**Authors:** Daniel Carvalho Pires Sousa, Washington Soares Ferreira Júnior, Ulysses Paulino Albuquerque

**Affiliations:** 1 Laboratório de Ecologia e Evolução de Sistemas Socioecológicos, Departamento de Botânica, Centro de Biociências, Universidade Federal de Pernambuco (UFPE), Recife, PE 50670-901 Brazil; 2 Universidade de Pernambuco, Campus Petrolina, Rodovia BR 203, Km 2, s/n – Vila Eduardo, Petrolina, PE 56328-903 Brazil

**Keywords:** Ethnobotany, Evolutionary ethnobiology, Medicinal plant, Cultural salience, Cultural importance, Spatial/temporal variation, Children’s knowledge

## Abstract

**Background:**

Measures of the importance of medicinal plants have long been used in ethnobotany and ethnobiology to understand the influence of social-ecological system factors in the formation of individuals’ differential knowledge and use. However, there is still a gap in empirical studies that seek to understand the temporal aspects of this process.

**Methods:**

To overcome this issue, we used the concept of the structural core of medicinal plants, a theoretical-evolutionary model, which argues that the importance of medicinal plant resources is related to the increase in individual and population fitness. It represents the set of the most effective and available resources that would treat the most common diseases in an environment. This composition of knowledge would be conservative over space and time. To test these questions, we hypothesized that the composition of the structural core remains constant during temporal changes in a social-ecological context, and that the composition of the infantile structural core (new generation) is similar to that of the adults (older generation). For 2 years, we tracked the structure of important medicinal plants among the same 49 residents of a community located in Vale do Catimbau in Pernambuco, Brazil. We also compared the importance of the medicinal plants among two different generations, children/adolescents and adults, in the same space/time context.

**Results and Conclusion:**

Our results refuted both hypotheses. Regarding the composition of important medicinal plants through temporal variations and for children's learning, our results were not predicted by the model. This suggests that the structural core should not be regarded as a conservative phenomenon, but rather a congenital, dynamic, and plastic occurrence that has adapted to configure itself as a short-term population response to the treatment of local diseases.

**Supplementary Information:**

The online version contains supplementary material available at 10.1186/s13002-022-00548-2.

## Introduction

The knowledge and local use of medicinal plants are characteristics of medical systems, which are systematically analyzed in ethnobotany and ethnobiology. Many studies consider this cultural domain fundamental to allowing subsistence societies to survive the different challenges present in social-ecological systems [1–4]. However, some studies on the structure of this knowledge have shown that human populations have a unique tendency to practice and recognize the importance of only a few sets of medicinal plants, despite the vast repertoire of culture that can be accumulate and pass through generations on the medicinal properties of local ecological resources [2, 5–7]. In this sense, our study starts with the objective of investigating whether there are intrinsic properties of the systems that influence the construction and conservation of this knowledge structure based on importance. We also seek to track the composition of the knowledge over time.

The importance of plant resources has been studied and measured in different ways in ethnobiology and ethnobotany. For example, Ferreira Júnior et al. [8] used the cultural salience index of species [9, 10] to identify the most preferred species for the treatment of inflammation. Bonifácio et al. [11] defined the cultural importance of certain plant species within the systems to elaborate and discuss priority conservation strategies using the use value index [12, 13]. Medeiros et al. [14] defined the most important knowledge using the relative importance index [12, 15] to discuss the differential use of medicinal plants according to their taste and smell. Characterizing the importance of medicinal plants in ethnobotanical research can also be defined via measures of cultural significance [6, 16, 17].

These varied ways of measuring the “cultural importance” of a particular medicinal resource show us how difficult it is to develop a definitive measure of the cultural and practical significance of a particularly renown taxon [18]. The cultural significance of certain elements in the system is a natural process of the structure of the populational knowledge of societies [19–21]. Therefore, all these analyses and scientific metrics only seek to significantly delimit those that researchers can define as relevant, or not, to formulate their investigations and work questions.

There is still a lack of studies in ethnobotany that seek to investigate the evolution of important knowledge over time, with some exceptions [22, 23]. These studies may take the form of tracking the components of this knowledge in punctual collections through space/time [such as through free lists or questionnaires] or investigating how many new generations [children and adolescents] “absorb” the knowledge of plant species from this structure. The measurement and characterization of important medicinal plants are, in a way, tied to the explanatory variables that make up the current social-ecological context. There may be important types of knowledge that are historically significant for the population studied. However, to our knowledge, virtually no study has conducted successive interviews to try to analyze the variation, or lack thereof, in the knowledge considered important by local populations. How should the body of the knowledge of important medicinal plants behave across space and time? Our study seeks to advance this critical question to enrich ethnobotanical theory and practice, using the concept of the structural core of medicinal plants as a theoretical-evolutionary model of the knowledge in medical systems [2].

The concept of the structural core, developed by Ferreira Júnior and Albuquerque [2], is based on the hypothesis that medicinal plants with high popularity [core] have adaptive traits that address the therapeutic needs in a medical system. Important medicinal knowledge is characterized as a cultural “basic kit” for dealing with diseases in the environment. Based on the literature, the authors argue that these plants become important because they have adaptive traits, represented by the plant’s efficacy in the treatment of diseases, availability in the environment, and treatment of common diseases, thereby providing the best available chance to speed up the cure of an illness that incapacitates the people in local communities [2]. This would allow the efficient “real-time” maintenance of local disease treatment processes. Thus, with the passage of space and time, in a non-stochastic ecological way the knowledge of these medicinal plants tends to accumulate in knowledge systems [24, 25]. Therefore, the selection of important medicinal plant species would be aimed at long-term conservation.

This hypothesis is supported by the adaptive memory model [26], in which individuals are genetically “tuned” to more efficiently memorize a behavior evaluated to be adaptive to the context [27–29]. In addition to this is the fact that local populations share the recognition of “prototypical” medicinal plants, which are those that are a reference for the treatment of common diseases, through mnemonic clues such as taste and smell [5, 30]. In this sense, important medicinal practices that involve the knowledge and use of medicinal plants that are perceived as more effective or available, and thus provide a greater advantage of fitness, would automatically or preparedly stimulate the synaptic connections selected over time to solve medical problems.

These two cognitive and cultural-referential structures work together to modulate the structural core in an increasingly “conservative” disposition across space and time, preserving intergenerational knowledge of the local medicinal plants that are most important in the treatment of diseases.

Other evolutionary theories, such as the theory of cumulative cultural evolution and natural pedagogy, can be used as a guide in explaining the adaptive importance of medicinal plants. The theory of cumulative cultural evolution [31] refers to the process through which cultural traditions are gradually modified and improved over time [32]. This process allows societies to “accumulate” solutions to problems faced by individuals that could not have been invented by a single person [33]. It also enables individuals to reach responses in a shared way, thereby distributing the efforts, risks, and time of the trial-and-error processes [32]. For example, let us consider the practice of using tea as a remedy for stomach aches. Boiling the water, selecting the medicinal plant parts, and determining the time required to extract the therapeutic properties via infusion are problem-solving behaviors that are not likely to be practiced by someone new to the idea of treating stomach pain-related diseases. This complex package of information about medical systems can accumulate in the systems and be passed on in blocks to new cultural learners. According to the theory of natural pedagogy, during childhood, children are the most favored in social learning. This is because, in addition to having cognitive mechanisms that allow them to learn social information quickly and effectively, adults tend to facilitate the learning environmental information by directly teaching the most adaptive ones [34, 35].

Thus, based on the assumptions proposed above, to investigate the temporal evolution of the important medicinal plants remembered by people, we hypothesize that (H1) the composition of the structural core remains constant during the temporal variation in a social-ecological context. We expect that (P1), concerning satellite plants [plants that are not part of the structural core], the composition of medicinal plant species from the structural core at Period 1 will be similar to the composition of species of medicinal plants from the structural core at Period 2. In addition, to analyze how much the new generations remember the medicinal plants considered to be important by the older generation, we hypothesized that (H2) the composition of the infantile structural core [new generation] is similar to that of the adults [older generation]. We expect that (P2), concerning the satellite plants, the composition of medicinal plant species in the infantile structural core is similar to that of the adult structural core.

## Materials and methods

### Characterization of the area and study sites

**Fig. 1:**
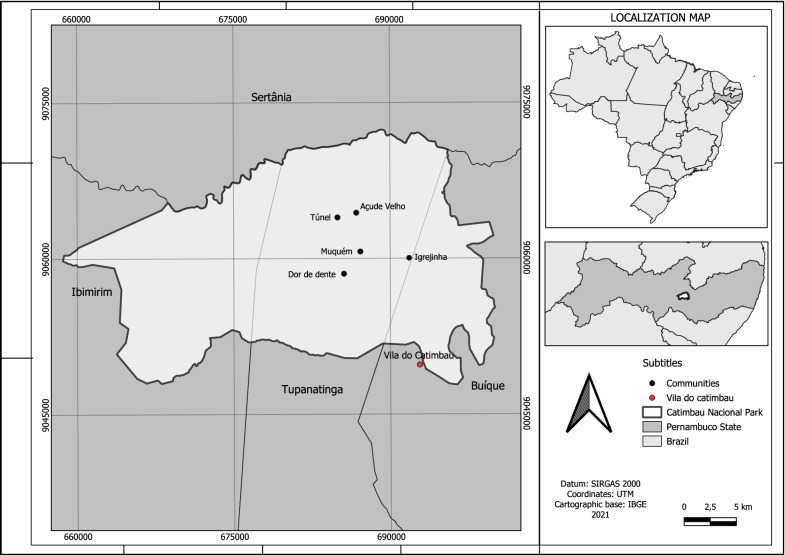
Location map of the territorial limits of PARNA do Catimbau, Vila do Catimbau, and the communities studied from 2017 to 2019

The study was performed within the National Park of Vale do Catimbau (PARNA do Catimbau; 8°32′54.2″ S 37°14′49.6″ W). This park is an Integral Protection Conservation Unit in the state of Pernambuco, in the northeast region of Brazil, which had its limits federalized on December 13, 2002. Consequently, the ecological resources of its interior are protected by law [36]. This unit covers an area of 64,294 ha and is located within the municipalities of Ibimirim, Tupanatinga, and Buíque (Fig. 1). The climate is classified as tropical semiarid with a Caatinga (Brazilian savannah) ecosystem, an annual average temperature of approximately 23 °C, and precipitation between 486 and 975 mm, with rainfall concentrated between March and July [37]. The nearest village and entrance to PARNA do Catimbau is Vila do Catimbau, which has approximately 2240 inhabitants [38]. There, small markets, building material stores, a health center, and an elementary school can be found. However, access to Vila do Catimbau is difficult because of the presence of precarious mud roads. Prolonged drought and lack of rain are hallmarks of this region.

There are at least 17 small communities distributed across PARNA do Catimbau [37]. All communities operate in subsistence mode as its residents collect resources from vegetation to use as natural remedies, firewood, and for building houses and fences. Some residents own small bean and corn plantations. Residents also raise grazing animals, such as goats and cattle, and backyard animals, such as chickens, for food or sale. Water is a scarce resource that keeps people within a permanent rationing system. There are no health facilities close to the communities inside the park; as a result, individuals with medical emergencies of any kind must be carried out in some way, either to the Vila do Catimbau Health Care or to the large urban centers of the nearby municipalities. Likewise, there are no schools of any kind inside the park; however, the children receive the assistance of the government for transportation to carry out their studies in Vila do Catimbau. In addition, some families within PARNA do Catimbau receive food, as well as educational, social, and citizen assistance from the non-governmental organization Amigos do Bem [www.amigosdobem.org], whose edifice is located at the entrance to Vila do Catimbau. This organization transfers goods and services that are deemed national and international financial aid for these residents.

Before making contact with any residents of the region, we met with the head of the Vila do Catimbau Family Health Post to explain our research objectives. We also asked for general information about their way of life, and the number of residents and residences located within the PARNA do Catimbau.

### Ethical and legal aspects

We spent several weeks building rapport within the local communities. All interviewees (> 18 years old) who agreed to participate in this study were invited to sign the Free and Informed Consent form. Concerning the interview with the children (aged 6 to 14 years), all responsible adults (> 18 years) were invited to sign a second Free and Informed Consent form authorizing the participation of the children. Additionally, for the children who agreed to participate, we invited them to sign (or place their fingerprint on) another specific consent form in keeping with the current legislation of the Ministry of Health (Resolution 466/12 of the National Health Council). This legally protects them by keeping their identities and shared information confidential.

This study was submitted and approved by the Official State Body of the Research Ethics Committee of the University of Pernambuco (CAAE:89887817.6.0000.5207).

### Ethnobotanical data collection

Data collection for this research was carried out in two periods; the first was conducted between January and November 2017, and the second was between January and April 2019. The initial contact was done with a team of researchers associated with the National Institute of Science and Technology in Ethnobiology, Bioprospection, and Nature Conservation (https://www.inctethnobio.com/) and the Laboratory of Ecology and Evolution of Socioecological Systems (https://www.evoethnobio.com). On this event, several weeks were dedicated to building the local people's trust in our research group.

For all participants interviewed (adults or children), we used the free-list method to record local the knowledge of medicinal plants [9, 12]. This method was chosen because, in addition to being semantically efficient in acquiring abundant amounts of information about local knowledge, it is simple and objective in its application, which makes it possible to maximize the number of interviews. The interview consisted of asking a single question, “Which medicinal plants do you know?” without additional stimuli. The responses allowed the order of recall of the medicinal plants per person to be recorded. As much as possible, conducting the interviews in an individualized manner was prioritized to prevent other people from stimulating crossed memories.

In total, there were five rural communities located inside the park where the interviews were conducted: Igrejinha, Muquém, Dor de Dente, Túnel, and Açude Velho (Fig. 1). All of them have similar forms of housing, and the residents had similar lifestyles among themselves and other communities located within the park. According to a survey by our group of researchers, Igrejinha has approximately 112 residents and 51 homes, Muquém has 38 residents and 21 homes, Dor de Dente has 13 residents and 7 homes, Túnel has 6 residents and 2 homes, and Açude Velho has 17 residents and 10 homes. We interviewed 49 residents from these five communities.

To test the first research hypothesis, we interviewed the same 49 participants (> 18 years old) in periods 1 and 2. Specifically, 33 participants were in Igrejinha, eight in Muquém, two in Dor de Dente, three in Túnel, and three in Açude Velho. To test the second research hypothesis, we focused on the community of Muquém and interviewed 25 adult participants [> 18 years old] and 18 child participants [aged between 6 and 14 years] in Period 2. It is important to note that we decided to consider children in this age group because ethnobotanical studies suggest that it is around this age that a person in a subsistence context in social-ecological systems begins to become “culturally apt” to develop and use the knowledge and practices of their society [17, 39].

In summary, to test the first hypothesis, we carried out free lists of medicinal plants with the same 49 participants at an interval of approximately 2 years (periods 1 and 2). To test the second hypothesis, we carried out free lists of medicinal plants for 25 adults and 18 children in the same period and community.

### Data analysis

All our predictions initially depended on the definition of which plants we will consider to be part of the structural core, in other words, which plants make up the set of important medicinal plants, and which plants are not regarded as part of it. As noted in the introduction, we will refer to non-structural core plants as “satellite plants.” Because Ferreira Júnior and Albuquerque [2] suggested that core plants have intrinsic adaptive characteristics that make them more “popular” within a local medical system, we decided to measure this popularity through the cultural salience index [9, 12], an analysis that takes into account both the frequency and the recall order of all the information recorded in the free lists. In this analysis, the more frequent and first remembered an item, the more salient [or important for the culture in question] it is.

However, this method of analysis results in a list of items on a decreasing numerical continuum, and any further attempt to divide this list into “structural core plants” and “satellite plants” would be subjective and could produce a biased interpretation of the results. To solve this impasse of objectivity, we used the statistical output defined by Chaves et al. [40], called the “salience threshold.”

Chaves et al. [40] developed a method of analysis that compares the salience list obtained in the field with 1000 salient lists generated in a statistical environment simulating a null scenario. For the null scenario to have the same characteristics as the real scenario in the field, these salience lists are based on hypothetical free lists that are randomly generated, according to the same number of participants, the same number of items cited by the population, and the same average citations per participant [40]. Using this statistic, salience values obtained from the field should be significantly different from those expected by the salience lists generated by the null scenario, with items that have higher or lower values than those expected by chance. In this sense, the “salience threshold” defines which items become more prominent when comparing the observed values of salience with the expected values against the null model, with a decision alpha of 5% [40]. This method of analysis allowed us to characterize the knowledge structure in structural core plants and satellite plants of the 49 people in periods 1 and 2, and of the 25 adults and 18 children from Muquém in Period 2.

After statistically defining the “salience threshold,” to directly test hypotheses 1 and 2, we used a permutational multivariate analysis of variance (PERMANOVA) [41]. The data matrices were constructed in Excel as follows: for Hypothesis 1, the rows presented each of the 49 participants from Period 1; this was followed by the same 49 participants, only now related to Period 2. The columns were composed of all the medicinal plants contained in the contextual structural core of periods 1 and 2. If the participant remembered any medicinal plant contained in the columns, we marked “1”; otherwise we marked “0.” The same procedure for the core plants was also performed for the construction of the related matrices for Hypothesis 2; however, instead of relating the participants' lines by period, we related them by “adults” and “children” [see Additional files 1, 2: data].

The Jaccard index was used to calculate the dissimilarity index using presence/absence data matrices. All dissimilarity indices were calculated using the vegdist function of the vegan 2.5–7 package in the R development environment [42]. The homogeneity of the multivariate dispersions was calculated to analyze the variation in species composition using the betadisper function of the vegan package [43]. For a visual analysis of the dissimilarity, we plotted the principal coordinates (PCoA) using the base plot function of R.

## Results

In the first period, the 49 participants recalled 81 medicinal plants. Using the “salient threshold,” we characterized the 15 most salient plants as the structural core, and the remaining 66 were regarded as satellites. In the second period, 2 years later, the same participants recalled 77 medicinal plants, 11 were characterized as the structural core and 66 as satellites (Table 1). The Jaccard dissimilarity index indicated that the composition of medicinal plants present in the structural core in Period 1 is significantly different from that present in the structural core of Period 2 (*p* < 0.001). These results refute our first hypothesis.

**Table 1:** Structure of knowledge of important medicinal plants from the 49 residents of Vale do Catimbau, 33 in Igrejinha, 8 in Muquém, 2 in Dor de Dente, 3 in Túnel, and 3 in Açude Velho [NE Brazil], in the years 2017 and 2019, characterized by a structural core and satellite plans after calculating the “salient threshold”

Knowledge structure in 2017				Knowledge structure in 2019			
Order	Popular name	Salience	*p* value	Order	Popular name	Salience	*p* value
**Structural core [*n* = 15]**				**Structural core [*n* = 11]**			
1	ameixa	0.4644	0.0000	1	ameixa	0.5347	0.0000
2	hortelã	0.4137	0.0000	2	quixaba	0.3373	0.0000
3	papaconha	0.3616	0.0000	3	papaconha	0.3193	0.0000
4	jatobá	0.3590	0.0000	4	aroeira	0.2977	0.0000
5	mastruz	0.3462	0.0000	5	jatobá	0.2803	0.0000
6	quixaba	0.3343	0.0000	6	mastruz	0.2584	0.0000
7	babosa	0.3147	0.0000	7	mororó	0.1929	0.0028
8	aroeira	0.3100	0.0000	8	alcansus	0.1790	0.0067
9	juá	0.2202	0.0001	9	erva cidreira	0.1690	0.0125
10	bássimo	0.2064	0.0006	10	babosa	0.1596	0.0228
11	umburana de cambão	0.1857	0.0029	11	bom nome	0.1477	0.0452
12	umburana de cheiro	0.1853	0.0030	**Satellite plants [*n* = 66]**			
13	alcansus	0.1636	0.0128	12	hortelã	0.1429	0.0579
14	federação	0.1620	0.0148	13	angico	0.1376	0.0760
15	mororó	0.1422	0.0459	14	umburana de cambão	0.1374	0.0766
**Satellite plants [*n* = 66]**				15	bássimo	0.1359	0.0820
16	baraúna	0.1391	0.0535	16	umburana de cheiro	0.1172	0.1861
17	velame	0.1249	0.1059	17	sassafrás	0.1153	0.2011
18	erva cidreira	0.1174	0.1468	18	quebra faca do sertão	0.1084	0.2595
19	jurubeba	0.1137	0.1701	19	algaroba	0.0882	0.4707
20	alecrim	0.1101	0.1970	20	capim santo	0.0832	0.4714
21	sassafrás	0.1065	0.2266	21	jurubeba	0.0794	0.4239
22	algaroba	0.1060	0.2308	22	juá	0.0779	0.4043
23	quebra faca do sertão	0.0956	0.3310	23	federação	0.0742	0.3605
24	arruda	0.0942	0.3454	24	jenipapo	0.0701	0.3128
25	bom nome	0.0918	0.3735	25	caju roxo	0.0677	0.2845
26	caju roxo	0.0825	0.5145	26	arruda	0.0673	0.2797
27	carrapixo de boi	0.0751	0.4242	27	baraúna	0.0614	0.2164
28	pau d’arco	0.0734	0.4026	28	velame	0.0564	0.1711
29	romã	0.0608	0.2556	29	eucalipto	0.0562	0.1697
30	maracujá do mato	0.0591	0.2379	30	catingueira	0.0532	0.1441
31	angico	0.0528	0.1754	31	jurema preta	0.0518	0.1325
32	sambacaité	0.0480	0.1330	32	pau d’arco roxo	0.0474	0.0986
33	jurema preta	0.0438	0.1017	33	alecrim	0.0445	0.0819
34	mandacaru	0.0429	0.0953	34	alento	0.0406	0.0627
35	moleque duro	0.0408	0.0818	35	jucá	0.0396	0.0587
36	melancia	0.0405	0.0802	36	capeba	0.0372	0.0476
37	eucalipto	0.0394	0.0746	37	pau de leite	0.0372	0.0476
38	catingueira	0.0378	0.0651	38	rabo de raposa	0.0371	0.0472
39	manjirioba	0.0374	0.0625	39	maracujá do mato	0.0298	0.0252
40	capim santo	0.0359	0.0555	40	melancia	0.0298	0.0252
41	capeba	0.0324	0.0415	41	sambacaité	0.0279	0.0207
42	quina quina	0.0314	0.0379	42	moita de mulher	0.0273	0.0192
43	marmeleiro	0.0305	0.0349	43	quebra pedra	0.0257	0.0164
44	rabo de raposa	0.0257	0.0219	44	alfavaca	0.0256	0.0161
45	cana de macaco	0.0246	0.0191	45	mulungu	0.0235	0.0132
46	jenipapo	0.0233	0.0167	46	manjirioba	0.0231	0.0125
47	canelinha	0.0231	0.0163	47	manjericão	0.0216	0.0104
48	ubaia	0.0188	0.0089	48	boldo	0.0204	0.0087
49	alfavaca	0.0179	0.0076	49	coroa de frade	0.0168	0.0047
50	vassourinha	0.0173	0.0070	50	facheiro	0.0165	0.0045
51	pau de leite	0.0169	0.0066	51	cambuim	0.0164	0.0045
52	beladona	0.0168	0.0065	52	canafistula	0.0159	0.0043
53	pau ferro	0.0168	0.0065	53	carrapixo de boi	0.0155	0.0041
54	alento	0.0152	0.0051	54	marmeleiro	0.0154	0.0041
55	cebola branca	0.0149	0.0049	55	batata de purga	0.0148	0.0036
56	moita de mulher	0.0143	0.0045	56	folha miúda	0.0148	0.0036
57	mulungu	0.0141	0.0044	57	ouricuri	0.0141	0.0034
58	erva doce	0.0136	0.0040	58	umbuzeiro	0.0141	0.0034
59	umbuzeiro	0.0126	0.0035	59	biratanha	0.0122	0.0028
60	mata pasto	0.0125	0.0033	60	canzenzo	0.0122	0.0028
61	plenito	0.0122	0.0032	61	cana de macaco	0.0102	0.0019
62	cravo	0.0117	0.0030	62	mijo de ovelha	0.0099	0.0018
63	ouricuri	0.0115	0.0028	63	romã	0.0099	0.0018
64	carcara	0.0105	0.0026	64	vassourinha	0.0097	0.0018
65	cambuim	0.0100	0.0023	65	espada	0.0094	0.0017
66	jucá	0.0087	0.0018	66	maracujá	0.0094	0.0017
67	folha miúda	0.0080	0.0016	67	melão de são caetano	0.0094	0.0017
68	canafistula	0.0055	0.0012	68	canelinha	0.0093	0.0017
69	batata de onça	0.0045	0.0009	69	louco	0.0089	0.0016
70	urtiga	0.0040	0.0008	70	cabeça de nego	0.0078	0.0012
71	coroa de frade	0.0036	0.0008	71	beladona	0.0068	0.0009
72	pau de alho	0.0036	0.0008	72	marcela	0.0068	0.0009
73	cabacinho	0.0035	0.0008	73	caroá	0.0056	0.0006
74	endro	0.0034	0.0008	74	pau ferro	0.0051	0.0004
75	quebra pedra	0.0034	0.0008	75	pau d’arco branco	0.0043	0.0004
76	biratanha	0.0033	0.0008	76	maçaranduba	0.0041	0.0003
77	agase	0.0030	0.0008	77	cabuci	0.0031	0.0002
78	pinhão brabo	0.0029	0.0007				
79	aveloz	0.0027	0.0007				
80	espinheiro	0.0011	0.0002				
81	maracujá	0.0011	0.0002				

**Fig. 2:**
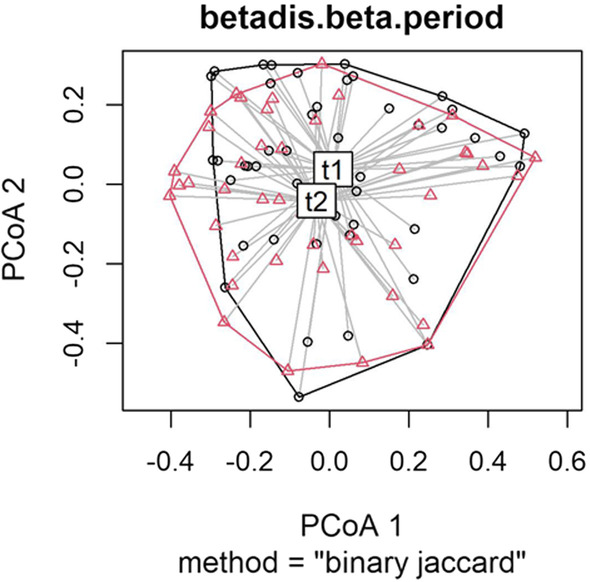
Visual analysis of the dissimilarity of the data from Hypothesis 1, resulting from plots of the main coordinates from Period 1 (t1) in 2017 and from Period 2 (t2) in 2019

**Fig. 3:**
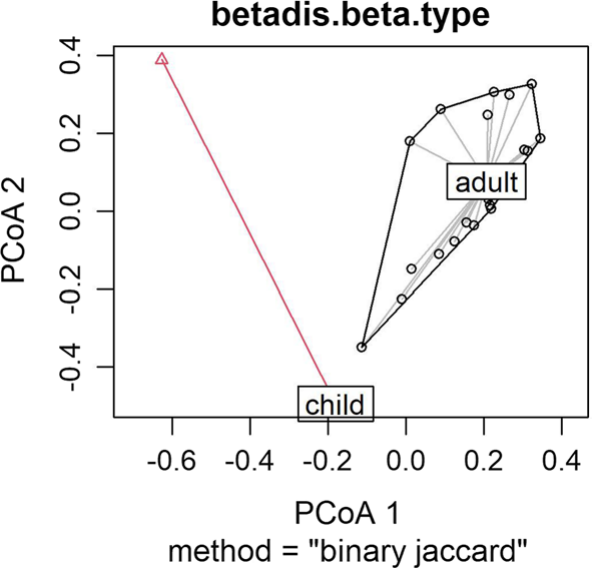
Visual analysis of the dissimilarity of the data from Hypothesis 2, resulting from plots of the main coordinates from adults and children in Period 2 in 2019

Regarding the second hypothesis, the 25 adults from Muquém recalled 53 medicinal plants, and, after the “salient threshold,” 9 were categorized as structural core plants and 44 were categorized as satellite plants. The 18 children recalled 22 medicinal plants, two of which were characterized as structural core plants and 20 as satellite plants (Table 2). The Jaccard dissimilarity index showed that the composition of medicinal plants present in the structural core of the adults was significantly different from that of the children (*p* < 0.001). Thus, these results also refute the second hypothesis. A summary of all the statistics reported above is presented in Table 3 for Hypothesis 1 and in Table 4 for Hypothesis 2. The visual analysis of the dissimilarity of both tests, resulting from plots of the main coordinates of our data, is presented in Figs. 2 and 3.

**Table 2:** Structure of knowledge about important medicinal plants of the 25 adults and 18 children living in the community of Muquém in Vale do Catimbau (NE Brazil), characterized by structural core and satellite plants after calculating the “salient threshold.” This information was taken in the same space/time context in 2019

Structure of adult knowledge				Structure of knowledge of children and adolescents			
Order	Popular name	Salience	*p* value	Order	Popular name	Salience	*p* value
**Structural core [*n* = 9]**				**Structural core [*n* = 2]**			
1	ameixa	0.7822	0.0000	1	ameixa	0.2698	0.0023
2	quixaba	0.5127	0.0000	2	capim santo	0.1817	0.0497
3	aroeira	0.3877	0.0000	**Satellite plants [*n* = 20]**			
4	jatobá	0.3794	0.0000	3	erva cidreira	0.1786	0.0542
5	bom nome	0.3342	0.0001	4	jatobá	0.1556	0.1005
6	algaroba	0.2851	0.0020	5	umburana	0.1095	0.2960
7	sassafrás	0.2731	0.0035	6	mastruz	0.0952	0.4211
8	juá	0.2452	0.0112	7	algaroba	0.0873	0.5467
9	bássimo	0.2399	0.0136	8	caju roxo	0.0833	0.5159
**Satellite plants [*n* = 44]**				9	goiaba	0.0635	0.3864
10	angico	0.1933	0.0708	10	limão	0.0556	0.3197
11	umburana de cheiro	0.1739	0.1233	11	quixaba	0.0540	0.3040
12	quebra faca do sertão	0.1724	0.1282	12	beladona	0.0476	0.1843
13	caju roxo	0.1717	0.1305	13	juá	0.0476	0.1843
14	umburana de cambão	0.1634	0.1643	14	mandacaru	0.0476	0.1843
15	baraúna	0.1354	0.3125	15	pinhão	0.0476	0.1843
16	erva cidreira	0.1102	0.5087	16	umbu	0.0357	0.1309
17	pau d’arco roxo	0.1029	0.4531	17	boldo	0.0317	0.1001
18	pinhão brabo	0.1013	0.4412	18	sassafrás	0.0286	0.0990
19	mororó	0.0853	0.3180	19	jenipapo	0.0190	0.0417
20	mastruz	0.0818	0.2932	20	aroeira	0.0095	0.0024
21	babosa	0.0796	0.2767	21	romã	0.0095	0.0024
22	piranha	0.0779	0.2650	22	laranja	0.0079	0.0000
23	papaconha	0.0760	0.2527				
24	capim santo	0.0717	0.2248				
25	mandacaru	0.0569	0.1361				
26	umbuzeiro	0.0508	0.1059				
27	alecrim	0.0465	0.0880				
28	catingueira	0.0460	0.0858				
29	rabo de raposa	0.0458	0.0850				
30	goiaba	0.0400	0.0638				
31	jucá	0.0375	0.0536				
32	jurubeba	0.0336	0.0413				
33	eucalipto	0.0320	0.0373				
34	velame	0.0320	0.0373				
35	chumbinho de areia	0.0300	0.0334				
36	canzenzo	0.0280	0.0285				
37	feijão brabo	0.0262	0.0250				
38	maracujá do mato	0.0243	0.0215				
39	arruda	0.0231	0.0197				
40	manjirioba	0.0200	0.0151				
41	biratanha	0.0176	0.0112				
42	canelinha	0.0169	0.0100				
43	pau ferro	0.0165	0.0098				
44	andu	0.0160	0.0094				
45	cabeça de nego	0.0154	0.0088				
46	jurema preta	0.0122	0.0052				
47	jiquiri	0.0080	0.0030				
48	marcela	0.0067	0.0020				
49	abacate	0.0064	0.0018				
50	araçá	0.0060	0.0015				
51	sucupira	0.0040	0.0006				
52	romã	0.0033	0.0004				
53	amargoso	0.0020	0.0003

**Table 3:** Permutational multivariate analysis of variance (PERMANOVA) results from testing the similarity of the composition of medicinal plant species that were characterized as structural core plants by the same 49 residents of Vale do Catimbau in the two interview periods of 2017 and 2019

	Degree Freedom	Sum Of Squares	Mean Squares	pseudo-F	R^2^	*p* value
Period	1	1.7986	1.79855	6.347	0.06604	0.001
Residuals	91	25.4353	0.27951		0.93396	
Total	92	27.2338			1

**Table 4:** Permutational multivariate analysis of variance (PERMANOVA) results from testing the similarity of the composition of medicinal plant species, characterized by the 25 adults and 18 children living in the community of Muquém in Vale do Catimbau (NE Brazil). These results were measured in the same space/time context in 2019

	Degree Freedom	Sum of Squares	Mean Squares	pseudo-F	R^2^	*p* value
Stage of life	1	2.7374	2.7374	14.12	0.29343	0.001
Residuals	34	6.5915	0.19387		0.70657	
Total	35	9.3286			1

## Discussion

As seen above, our results indicate the complete opposite of the evolutionary model of medical systems based on the concept of a structural core that was predicted by Ferreira Júnior and Albuquerque [2]. The composition of important medicinal plants is not supported by the assumptions of specific adaptive long-term factors. Other more dynamic factors must also be considered. Our results allow us to affirm that the composition of plants from the structural core is not in a constant adaptive search for crystallization in the memory and recall of individuals in free lists. Moreover, at the population level, it has a very “volatile” lifespan from a temporal point of view. Nevertheless, we can also say that the importance of medicinal plants can be completely dependent on the current and historical temporal contexts.

### Short-term temporal adjustment?

According to the structural core model [2], the composition of medicinal plants considered most important by people is the result of medical system processes that involve efficacy, availability, the type of disease treatment, and social learning about plant resources. Therefore, while these factors are very important for the formation of the structural core, the change in the composition of plant species over time may be influenced by other factors of the system.

Several studies in the literature show how these processes can modulate behaviors that involve the knowledge and use of the plants considered to be important. Casagrande [30], for example, showed that factors such as availability and frequency of use, as well as the social and family roles that a person plays in society, directly influence their therapeutic choices. In a meta-analysis on the use value of useful plants, Gonçalves et al. [44] showed that factors, such as the local availability of plant resources, influence the differential use of firewood. This is because people may deliberately choose to use the plants available within their environment over others. Despite some opposing evidence [45], knowledge about medicinal plants of greater use value seems to be unaffected by phytosociological vegetation metrics [44]. Santos et al. [46] showed that plants considered important for different uses in the treatment of common diseases are strongly related to people's perception of them as effective for the treatment or cure [6, 47, 48].

The structural core model predicted that these important medicinal plants assemble all the necessary characteristics to act as protagonists in the treatment of diseases in medical systems, because they are perceived as most effective and/or available. It was expected that this set of information would accumulate over the long term, thereby creating a referential “basic kit” for the population over time.

However, as evidenced by our results, the structural core hypothesis was refuted. Such a “basic kit” did not exist, at least in the sense of targeting the specialization of medical systems through long-term cultural evolution. Perhaps we were not able to detect any pattern due to our time frame of 2 years of analysis. Although the structural core hypothesis was refuted, we believe that the only way to answer this question would be to invest in future long-term longitudinal studies to verify patterns in the composition of plant species considered important for the treatment of diseases over time.

Our findings indicate that the composition of the structural core is significantly more dynamic than that predicted by the model. It is always good to remember that most ethnobotanical studies, and socio-cultural and ecological data collection, are carried out at a specific point in space and time. Therefore, the 2015 model did not consider that the formation of the composition of important medicinal plants must have processes aimed at solving short-term spatial/temporal context challenges in social-ecological systems, in addition to those for long-term ones. Different disease episodes could greatly affect an individual’s patterns of remembering and using medicinal plants in daily life [49–51]. Our results indicate that there must be zones in the knowledge structure of medicinal plants that have very flexible compositions in the face of temporal variations.

The first question we can ask about this result is, “What are the factors that modulate this non-conservative temporal plasticity?” The second is whether a dynamic and plastic structural core adds an adaptive advantage to social-ecological systems. Furthermore, “Is the composition of important medicinal plants a contingent context-dependent phenomenon in medical systems?” Answering these questions will not be easy because there are many dimensions of systems to consider (such as socio-environmental, ecological, socio-cultural, and political) [52].

We believe that the explanation of our results is related to the hypothesis that important medicinal plants, based on their structural core, are knowledge structures created as a result of solving everyday short-term medical challenges. Therefore, the importance of medicinal plants must be a contingency phenomenon. The only way to test this hypothesis would be to carry out long-term studies with replicated experimental designs aimed at investigating the factors behind the formation of important medicinal plants both in context and over time. Although our study initially discarded the idea of the “basic kit,” it is expected that clear patterns may be found with 5 or 6 years of annual follow-ups. Only then will we be able to confirm or deny questions such as if there are conserved zones of the structural core with significant variations in the composition of important plants. The natural dynamics of ecological availability and the epidemiological context may be guiding the species composition of important plants.

Thus, to understand whether ecological availability is somehow related to the plasticity of the structural core over time, in periodic collections we must investigate the location and quantity of each of the important medicinal plants. Will they be the ones that are most commonly found in people's medicinal backyards? Will they be the most abundant ones in adjacent or exploited forests? Are they fully available to the community? Or are they anywhere near the studied communities? The fundamental method would be to simultaneously collect the same response variables (e.g., structural core and local preference) and explanatory variables (e.g., ecological availability) to investigate the patterns of the context, and also collect the minimum measurements of the same factors in the same way annually in long-term ethnobotanical research.

Epidemiological context can also be one of the main factors of medical systems in the modulation of a short-term adjusted and plastic structural core. In the original model of the structural core [2], it is argued that the composition of the most popular medicinal plants reflects the long-term evolutionary process in the treatment of common local diseases. Therefore, this “adjustment” of the composition of plants would be directed toward diseases that are always present in social-ecological systems. Therefore, if this composition adjustment is long term, 2 years of space/time variation should essentially capture the same set of recalled medicinal plants. However, our data suggest the opposite. We believe that what explains the plastic variation in the structural core, as captured by our measurements and relative to the local epidemiological context of the population at the time of the interviews, is how common the disease in question is [is it historical, seasonal, or newly established] and how frequently medicinal plants are used for their treatment.

Nevertheless, we must consider that, in addition to frequency, diseases have a specific severity level and latency period associated with them [3, 53–55]. Despite the severity of diseases inducing a low total richness of the knowledge of medicinal plant species for their treatment, because people tend to experience fewer new cure alternatives for illnesses with a high risk of death, these “idiosyncratic” medicinal plants may be related to the specific therapeutic resilience of the interviewed individual [54]. Furthermore, previous experience with these types of diseases can affect the individual’s recall rate of important medicinal plants.

Another variable of medical systems associated with the epidemiological context, which is the frequency of the use of plant resources, may also explain the plasticity of our results. Whether the diseases are common or uncommon, it is expected that the frequency of use of plant resources will indirectly influence the composition of the structural core. Casagrande [30], for example, directly verified the influence of the frequency of medicinal plant use on the formation of the composition of important medicinal plants [measured by salience]. In this way, Casagrande empirically showed that the more the plant is used, the more remembered the plant becomes. Priority is given to remembering those plant resources that are re-experienced daily or are significantly important to the person interviewed [49–51]. Reyes-Garcia et al. [51], however, showed that we can have plants rarely reported in interviews that are frequently used, and plants that are often reported in interviews and not used much. Our point here is that the frequency of use of medicinal plants in episodes of diseases may be modifying the rate of medicinal plant prioritization in a way that is not expected by the theoretical models of the structural core.

Thus, it is expected that over a 2-year period, individuals and populations may have several episodes of different diseases, which may be common or severe and historical or emerging. Moreover, the frequency of use of medicinal plants in each type of treatment varies. Accumulatively, this is what we believe may be influencing the plasticity of the structural core composition over time. Future studies that seek to better understand the short-term evolutionary dynamics of the composition of important plants, from the perspective of the frequency and severity of diseases, and the frequency of plant species use, could investigate measuring these epidemiological variables through methods such as residential therapeutic calendars, a survey of health posts, and individual perception [56].

### Medicinal plants considered important to adults are not the same as those considered important to children and adolescents

Another explanatory hypothesis of the functioning of the structural core model proposed by Ferreira Júnior and Albuquerque [2] is that adults are the fundamental learning pathways for the consolidation of a child’s knowledge about important things in the environment. As the structural core would be the population reference for adaptive information to deal with diseases that circulate in social-ecological systems, it was expected that important medicinal plants among adults would also be the most important among the younger generations of the community. However, our similarity data refuted this hypothesis. The first question we can ask regarding this is why medicinal plants considered important by adults do not seem to exert a referential influence on those seen as important for children. It is important to point out that it is more plausible to consider that children seem to share knowledge that is different from that of adults [57, 58]. In addition, it is noteworthy to mention that we do not have the effect of the influence of the factors mentioned above for the first hypothesis [temporal variation], because all the interviewees, children and adults, were inserted in the same social-ecological space/time contexts.

We must ask ourselves, “How do children learn and reason about medical and medicinal plant knowledge through their development in social-ecological systems?” The formation of the memory of children has already been widely debated in the recent literature on cognitive psychology [59, 60]. Nevertheless, for many decades, the study of children's cognition has always been neglected in scientific ethnobotany/ethnobiology discussions, based on the assumption that children are mere assistants in the process of acquiring information from the environment. However, recent studies have shown that the knowledge of children can be specific to their day-to-day activities and, when viewed from a group perspective, may even have a quality of their own as a type of “children's culture” [57, 58]. Empirical research also suggests that the prevalence of vertical transmission can be overestimated in self-reports about the learning model [57]. Moreover, because children learn a lot from other children, this indicates the importance of horizontal transmission in the formation of knowledge and child practice [61].

Several ethnobotany studies have discussed the role of parents and guardians in the cultural learning of children and adolescents. These studies have shown the importance of this vertical information transmission route for the formation of knowledge among young people [39, 57, 62]. They have also shown that medicinal plants that are either easier to use or easier to find close to homes are generally those that are incorporated into the knowledge of children, whereas medicinal plants from the forest are better known by adults [63]. It is worth noting that the knowledge of children is closely associated with specific aspects of social-ecological systems in which children and adolescents develop themselves. Examples of such are the different societies in which important knowledge and practices of medical systems are learned early on by children and teenagers [39, 64]. This suggests the role of adult facilitation in the cultural learning of valuable information. Notably, there is also the deliberate action by local healers of not sharing all the crucial information they know about their medicinal skills with younger healers. This is done to maintain their status as healer gurus who “know about all plants,” and, in doing so, they seek prestige and future consultations [65].

If we consider the evolutionary point of view of the acquisition of cultural knowledge and practices, compared to our phylogenetic cousin primates, humans develop the competencies of cultural skills long after weaning and the age of first reproduction [66]. This means that, compared with our relatives the primates, we have a very long childhood. This phenomenon is very costly from an energy perspective, as it depends on extensive support in food and care from several other members of the society [relatives or not] [66]. Nevertheless, it is seen in the literature that it is fundamental to allow long periods of social learning about the numerous codes and signs within the culture. This creates more episodes between the search for testing hypotheses, innovations, and learning to imitate the ability of adults in survival and reproduction [67].

The above-mentioned discussion shows that cultural learning in childhood goes through different stages in its development. On the one hand, it is heavily influenced by adult references who are relatives, as well as by pre-established rules of gender and social dynamics in places where children and adolescents grow up [63, 68, 69]. On the other hand, they have the autonomy to actively search for solutions and acquire knowledge about things in the environment to share with their peers between games and other joint activities [62]. Our results on the non-similarity of important medicinal plants between children and adults in the studied community may reflect a child’s long process of acquiring knowledge and cultural practices. This process is marked by questions of trial-and-error, testing of hypotheses, and imitation, which is characteristic of this phase of development. These results may also relate the factors related to the act of children sharing aspects of a culture [67, 69]. In children culture, the factors for considering the importance of a medicinal plant may have nothing to do with those considered by adults.

As a brief case study, of the plants contained in the structural core of children and adolescents in our community, only two medicinal plants were categorized as those that are a part of the children’s structural core; these are “ameixa” and “capim santo” (lemongrass). “Ameixa” is the medicinal plant with the highest importance among adults, both in 2017 and 2019 if we consider our entire sample. It is also the one with the highest importance among the adults residing only in Muquém, which would explain why it also appears as the plant with the highest importance value among children. However, “capim santo” is the 24^th^ most important medicinal plant among the adults of Muquém, which falls far from the first nine that make up its structural core (Table 2). The explanation for this pattern may be related to the fact that this medicinal plant is exotic, taken as a tea, and is popularly known in the region for the treatment of stomach complications, especially for children as it is considered “weaker.” Perhaps, the treatment events or perceived efficacy of these two plants are more common in communities related to children, especially in the administration of “capim santo” tea.

## Limitations and final considerations

Social-ecological and adaptive evolution is complex because these systems are a direct result of the cognitive, socio-cultural, and environmental/ecological systems of other complexes [52]. Our premises suggest that the popularity of important medicinal plants, measured by salience (structural core), is an adaptive pattern directly related to the simultaneous interaction of the cognitive (adaptive memory), social-ecological [prototypicality], and ecological availability system factors, adjusted to the short-term solution of the treatment of local diseases. It is also important to note that the free list is a method of collecting ethnobiological data that sometimes depends on the context in which the tool was used (see [70]). Our results also point in this direction. In light of this, future studies should gather information about the epidemiological contexts of communities. They should also analyze the role of other related variables, such as organoleptic properties, and relate them to tracking the importance of medicinal plants over space and time to advance the discussion above. Furthermore, ethnobiology and ethnobotany could carry out more longitudinal tracking analyses through the space and time of those variables and verify other factors related to the knowledge of children, such as age, gender, and diseases treated daily. This would be done with the aim of understanding this phenomenon of importance since the beginning of human development.

## Supplementary Information

**Additional file 1.** Raw data of the hypothesis 1.

**Additional file 2.** Raw data of the hypothesis 2.

## Data Availability

The datasets generated and/or analyzed during the current study are available from the corresponding author upon reasonable request.
